# Preliminary Studies of the Durability of Tools Used to Form Ceramic Tiles Made of Hardox 600 and NC11LV Steel

**DOI:** 10.3390/ma14051262

**Published:** 2021-03-07

**Authors:** Jan Marzec, Marek Hawryluk, Marcin Rychlik, Marzena M. Lachowicz, Maciej Suliga

**Affiliations:** 1 Department of Metal Forming, Welding and Metrology, Wroclaw University of Science and Technology, Lukasiewicza Street 5, 50-370 Wroclaw, Poland; jan.zbyszko.marzec@gmail.com (J.M.); marek.hawryluk@pwr.edu.pl (M.H.); marzena.lachowicz@pwr.edu.pl (M.M.L.); 2 Faculty of Production Engineering and Materials Technology, Czestochowa University of Technology, Al. Armii Krajowej 19, 42-201 Czestochowa, Poland; maciej.suliga@pcz.pl

**Keywords:** durability of tools, ceramic tiles, 3D scanning method, wear, resistant to intense abrasive wear

## Abstract

The study performs a comparative analysis of the wear of tools made of two wear-resistant materials: steel Hardox 600 and NC11LV, used in the process of forming a band for roofing tiles. The analyses were to allow the assessment of the possibility of replacing the standard material for tools in this process with a much less expensive tool steel for cold work after heat treatment (with a large number of carbides), as an alternative material dedicated to tools resistant to intense abrasive wear. The performed investigations included a macroscopic and geometrical analysis with the use of 3D scanning, microstructural analyses conducted by means of a light microscope, as well as an analysis of the topography of the working areas of the tools with the use of SEM, and microhardness tests. The obtained results demonstrate that the tools made of both materials were characterized with a similar level of wear, which, in the most critical area, reached over 4 mm, while the tools made of steel NC11LV worked over a much longer period of time without regeneration, equaling 912 h, and an insert made of steel Hardox 600 operated for 384 h. A higher tool life in the case of NC11LV steel may be the result of higher hardness and the presence of hard carbides.

## 1. Introduction

At present, the most frequently applied roofing materials include ceramic tiles, concrete tiles, steel tiles and bituminous tiles [1]. In the analysis of the application and parameters of all the roofing materials, the most optimal ones are still ceramic tiles, owing to their very long life, natural origin, the possibility of being used on roofs with a minimal slope, etc., and this simultaneously affects the development of the ceramic industry [2]. Currently, the main aspect of the development of this area is environmental protection [3] and recycling of waste [4], the search for new alternative materials for ceramic tiles [5,6] and the optimization of materials used in the production of machine elements for the production of tiles [7]. Elements mounted in the machines and devices on roofing tile production lines have to be especially resistant to abrasive wear, which occurs as a result of a contact with the extruded band of clay. At present, for the extrusion of a clay band for roofing tiles, horizontal band stamping presses are used, on the end of which a pug mill is placed, tipped with a so-called extrusion die, which is usually equipped with a set of two inserts, i.e., tools forming the final shape of the band [8,9,10]. Band stamping presses process masses with a humidity of 20–22% under the pressure of 2–10 MPa. This creates high pressures on these tools, causing their intensive wear and an increase in the temperature in the clay band–tool contact, as well as other destructive mechanisms and phenomena.

The wear process of this kind of element is affected by many, often contradictory, factors, which make the analysis of their operation very difficult and complex, similarly to the case of other shaping tools which work under extreme conditions (varying pressures and temperature gradients, a long path of friction, etc.) [11,12,13,14]. It is estimated that, under industrial conditions, the machines and devices are subjected to wear processes of the surface area, as a result of which 50% of elements become worn in the abrasive process, 15% as a result of adhesion and 8% due to the effect of erosion [15]. An analysis of the literature suggests that there are a few forms of abrasive wear. For example, during the movement of the loose abradant tangentially to the element’s surface, the abradant particles can deform the metal in an elastic or plastic way (forming grooves), or by way of micromachining [16]. Each of these operations depends on the hardness of the particles, their structure and the form of the grains. If, as a result of the operation of the abradant, reinforcement of the bottom of the grooves takes place after the material’s abrasion, this causes an increase in the metal’s resistance to further operation of the abradant. In the case when no reinforcement mechanism is observed, the metal begins to spall after its repeated plastic deformation [17,18]. Further, the structural changes should be considered in a situation when the abradant and the working areas move at a great speed in respect to each other. The heat of friction, in such a case, can significantly affect the conditions of abrasive wear and thus cause changes in the internal structure of the material. This can reveal itself, for example, as a local and zone-oriented lowering of the mechanical properties (hardness, yield stress, impact strength), thus leading to premature wear of the element. The application of elements made of wear-resistant materials makes it possible to prolong the operation time of this type of tool, thus prolonging the periods of time between the consecutive repairs.

Currently, among the modern materials used, e.g., for tools applied to extrude clay bands (whose main characteristic is high abrasion resistance), we can distinguish between a few groups: abrasion-resistant steels (Hardox, XAR, RAEX), boron steels, manganese steels (Hadfield) and the popular tool steels used for cold quenching and tempering. In the case of the relatively expensive wear-resistant steels, they are usually used as the base of the working element, i.e., for parts of elements which undergo a relatively less intensive wear [19,20,21,22,23]. In turn, in the case of boron steels, the boron (the main, but also expensive, alloy element) dissolves in the austenite, which makes it possible to obtain a bainitic structure with significantly refined grains already with regular hardening. Bainite, compared to martensite, is characterized by more favorable mechanical properties, including a 20–30% higher abrasion resistance [24]. In turn, manganese steels (containing as much as 12–13% manganese) are characterized by a very high tendency for reinforcement during operation, as micro-twins are formed in them under the effect of the cold work. Unfortunately, a problem in the case of these steels (in fact, cast steel in the case of steel Hadfield) is their workability, and thus the necessity of applying special processing tools becomes apparent [25]. That is why a certain alterative, especially in the aspect of the required mechanical properties in terms of the price, are the previously mentioned tool steels. Among the high-alloy steels for cold operations characterized by high wear resistance, we can list steels NCWV, NC11 and NC11LV, which owe that property to a high content of chromium [26,27,28]. Further, these steels do not contain the so-called pure carbides but rather mixtures of carbon and metallic elements. For example, steel NC11LV can contain the following carbides: (Cr, Fe)_3_C (due to the Fe_3_C carbides, in which some iron atoms can be replaced with chromium), (Cr, Fe)_7_C_3_ (due to the Cr_7_C_3_ carbides, in which some chromium atoms can be replaced by iron) or (Cr, Fe)_23_C_6_ (due to the fact that, in the Cr_23_C_6_ carbides, some chromium atoms can be replaced by iron). The hardness of steel can be increased through the use of, e.g., various thermo-chemical surface hardening methods, although it owes its high hardness mainly to numerous precipitations of secondary and primary, very hard, carbides [24,29]. Therefore, steel NC11LV can significantly exceed 600–850 HV (under special conditions, the steel reinforces itself as a result of deformation), which makes it proper for tools (forming the clay band) which are highly resistant to abrasive wear [30,31]. The literature contains a high number of studies referring to investigations and analyses related to the issue of machine component wear, including the aspect of the analysis and selection of different material solutions making it possible to increase the durability of this type of tool [32,33]. On the other hand, there is not much attention devoted to tools used in the processes of roofing tile clay band extrusion [30,31,32,33,34,35].

That is why advanced research in this field aiming at analyzing the premature wear of tools exposed to intensive abrasive wear, as well as proposing ways and methods of their improvement, is highly justified, as the mentioned wear constitutes a big problem, both in the scientific and the economical aspect.

The study performs a complex comparative analysis of selected tools, the so-called inserts, used to form the band for ceramic roofing tiles made of two different materials: Hardox 600 steel and an alternative material, NC11LV, in order to determine the causes and mechanisms of their wear.

## 2. Materials and Methods

**Figure 1:**
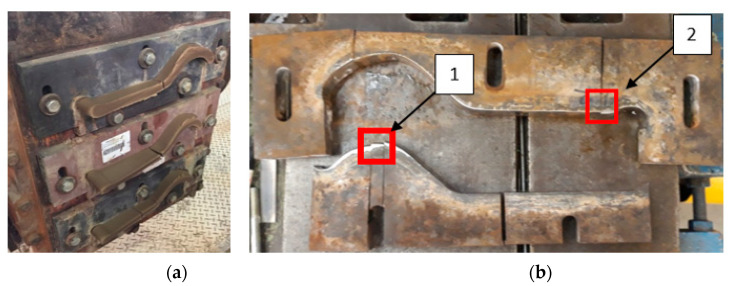
Photographs of: (**a**) inserts forming the clay band during operation; (**b**) upper and lower tools with marked areas from where test samples made of steel NC11LV were collected.

Figure 1 shows a photograph of tools used to form a clay band in the process of manufacturing ceramic tiles. The inserts are elements which are especially problematic due to the intensive wear of their working areas, which are in contact with the band of the formed clay. The materials used for the tools are characterized by high abrasion wear.

The standard tools were made of a 35-mm-thick Hardox 600 steel plate as-delivered, which did not require additional heat treatment. Hardened steel of the NC11LV grade was selected for the second set of tools. Quenching followed by tempering made it similar to Hardox steel and provided abrasion resistance in direct contact with the processed material. For Hardox 600, the measured hardness for the new tools was about 600–620 HB. NC11LV steel tools were subjected to the quench hardening process at the temperature of 960–1030 °C and then tempered at the temperature of 180 °C. This made it possible to obtain a hardness of even over 800 HV1. In order to perform a complex analysis, tests were carried out, which included the following:

Process and operation time analysis. The tool temperatures were measured with a pyrometer (Testo 845 pyrometer, Testo Poland, Pruszkow, Poland) and a thermal imaging camera (FLIR T530 camera, FLIR Systems, Inc. Wilsonville, OR, USA). The times of subsequent operations (the speed of extruding the band) were determined with the use of a high-speed camera (Casio Exilim Pro EX-F1, Casio, Tokio, Japan) capable of recording over 1000 frames per second.

Macroscopic analysis of the highest wear areas, performed by means of a Cannon EOS 50D (Canon Inc., Tokio, Japan).

3D scanning microscopic tests. For the assessment of the change in the geometry of the working surfaces, the measuring arm ROMER Absolute ARM 7520si integrated with the scanner RS3 (Hexagon Manufacturing Intelligence, Aarau, Switzerland), together with the Polyworks software 2015, was applied, making it possible to perform scanning in the real-time quality meshing technology.

Chemical composition analysis, carried out with the use of the LECO 750-GDS-QDP spectrometer (LECO Corporation, St. Joseph, MI, USA) with a glow discharge in an argon shield.

Microstructural tests, carried out by means of a metallographic microscope by Leica model DM6000M (Leica Microsystems, Wetzlar, Germany). The test samples were cut out from the inserts coming from the zones in which the wear was especially visible, with the use of an abrasive water jet to limit the effect of heat on the material’s microstructure and properties. The prepared samples with the proper dimensions were then subjected to the process of grinding and polishing on a grinding–polishing machine, Stuers350 (Struers, Copenhagen, Denmark), and then they were etched in a 3% nital solution.

Topography analysis. In order to perform an SEM analysis of the tool surface, the Tescan Vega 3 Scanning Electron Microscope (Tescan, Brno—Kohoutovice, Czech Republic) using a BSE (backscattered electrons detector) was used.

Hardness measurements. Tests were performed with the use of a LECO LC100 hardness tester (LECO Corporation, St. Joseph, MI, USA) and by means of the Vickers method, separately for each half of the insert. The first measurement was made at the edge of the insert responsible for the shaping of the clay band, and the following ones were carried out every 1 mm into the material. To obtain the most reliable results, the measurements were made in 10 points under the load of 1kgf (HV1). The test samples were prepared in a similar way to that in the case of the microstructural tests.

Impact toughness. The tests were performed on samples with a V-notch made according to the EN 10045-1 standard and realized with a Charpy hammer (KV 150) (Zwick Roell Group, Ulm, Germany). Two tests were performed for each tool for the upper and lower inserts, for both materials at a temperature of 20 and 40 °C (tool working temperature).

A comparative analysis of the wear of tools made of two wear-resistant materials: steel Hardox 600 and NC11LV, was conducted, which made it possible to assess the possibility of replacing the standard material for tools in this process with a much less expensive tool steel for cold work after heat treatment.

## 3. Results and Discussion

### 3.1. Process Analysis and Service Life

**Figure 2:**
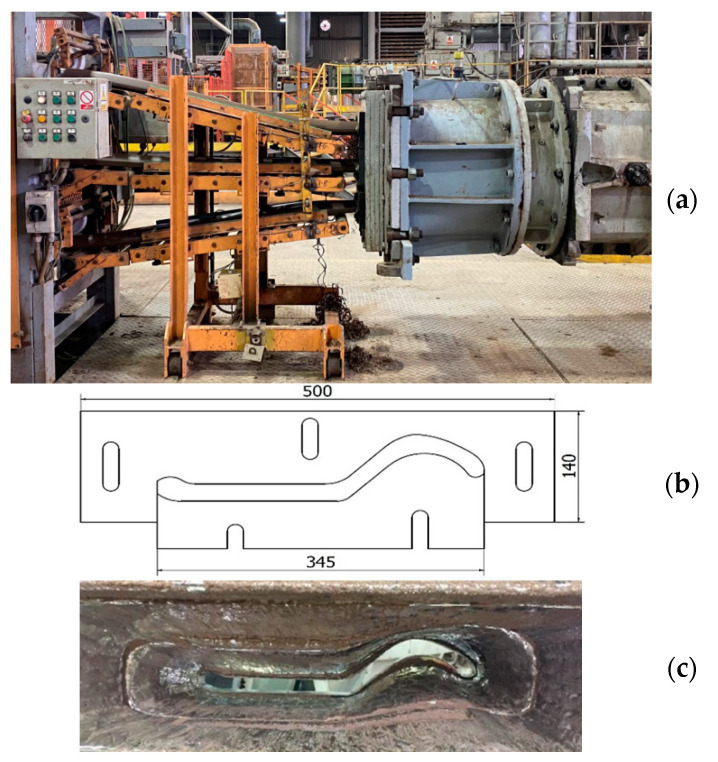
The process of extruding the clay band onto the ceramic tiles: (**a**) main view of the machines; (**b**) CAD drawing of a single set of tools with approximate dimensions; (**c**) photo of the worn chamber (mouthpiece).

Figure 2 shows the band shaping process. The photo (Figure 2a) shows (from the right) the head (mouthpiece) of the press (screw press) and three bands (top, middle, bottom), which are guided by a cutting belt on a three-segment stamp press. In turn, Figure 2b shows a diagram of both tools with their main dimensions, and Figure 2c shows a photo of the inside of the extruded press chamber for one of the three forming cavities.

**Figure 3:**
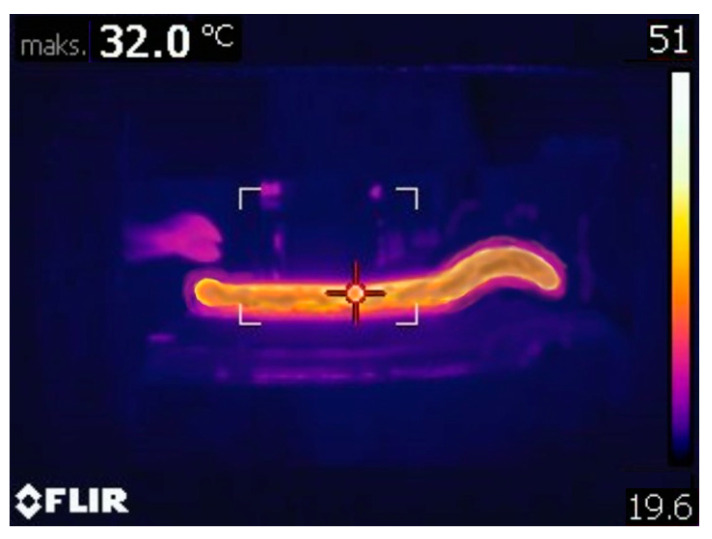
Thermovision analysis of the clay band and tool set.

Basically, the fragment of the entire technological process shown in Figure 2 is one of the key stages in the production of ceramic tiles and is carried out in four sub-processes. These include the following: final homogenization of the mass in a two-shaft mixer, de-aeration of the raw material in a vacuum chamber, squeezing the de-aerated strand through the screw press and finally cutting it. The main task of the tools is to provide the shape and thickness of the strand; therefore, special attention should be paid to these elements because the degree and type of wear affect the quality of the final product. During the extrusion process, the speed of the extruding clay band is about 0.3 m/s and the average temperature of the clay band is about 30–33 °C (Figure 3).

This temperature is caused by friction as a result of its being compressed in the band stamping press and in the pug mill, while, on the surface of the edges of the inserts, the temperature is several degrees higher as a result of additional friction and amounts to over 40 °C. This temperature was observed during the entire production for both tool sets. The higher temperature on the tools should positively influence the impact strength, which, in this type of process, can increase the tool life.

The analysis of the working time of the tool sets selected for the tests showed that the tools made of Hardox 600 steel worked for 384 h, while those made of NC11LV steel worked over 912 h. In both cases, the tools worked until their maximal acceptable wear (geometrical changes) which enabled the proper extrusion of bands being within the dimensional tolerances and the assumed quality (no visible surface defects). The quality of the surface of the tools and the squeezed strip was checked every 24 h and was based on a visual assessment and measurements of the key dimensions of the strip. The results show that the tool set made of NC11LV steel has an almost 3 times longer service life.

### 3.2. Macrospopic Analysis of Tools

**Figure 4:**
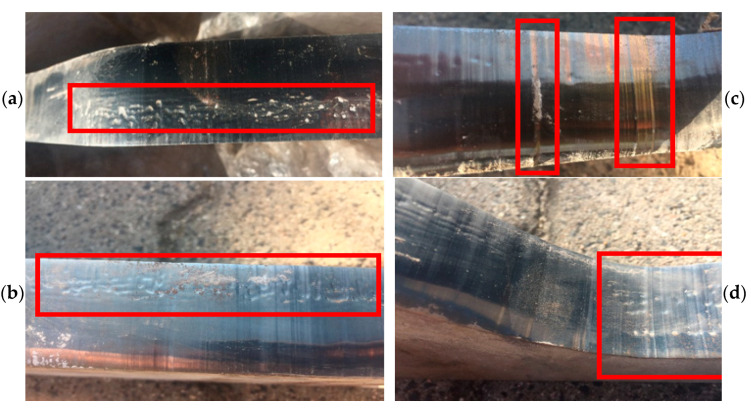
Macroscopic analysis of selected inserts forming the clay band: (**a**) visible traces of imprints; (**b**) scratches and cracks; (**c**) perpendicular scratches; (**d**) plastic deformations.

The macroscopic analysis (Figure 4) of the tools made of both materials demonstrated that their wear was similar. Numerous scratches were observed both in the direction of the extruded band (Figure 4b) and in the perpendicular direction (Figure 4c), as well as traces of imprints (Figure 4a) and cavities resembling plastic deformations (Figure 4d). The photographs presented above coming from the macroscopic analysis of the selected tools made of both materials show typical defects observed in the process of clay band extrusion.

They result from the difficult operation conditions, as such a band often contains hard particles, whose presence is a result of adding milled remains of burnt-out tiles, being one of the components, into each charge assigned for the band extrusion. An effect of the operation of the abrasion grains is the formation of scratches on the inner surface of the insert. The formed scratches can be reflected on the surface of the clay band, which can constitute a significant defect of the ready product. In the case of the presence of deep scratches on the inner surface of the inserts, it is necessary to grind them, so that they can be further applied.

### 3.3. Geometric Analysis by 3D Scanning Method

**Figure 5:**
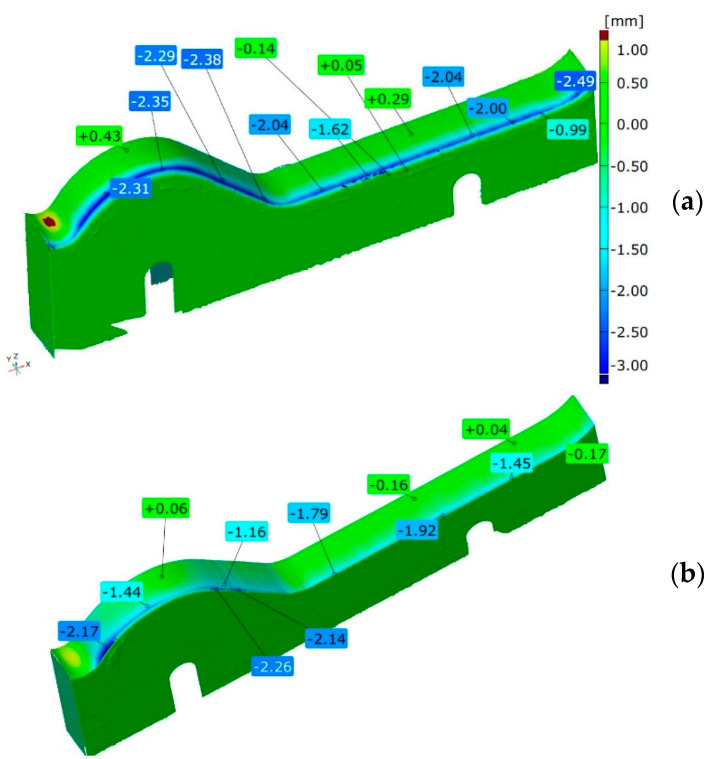
Results of scanning the lower tools made of steel: (**a**) Hardox 600 and (**b**) NC11LV.

The following stage of the research was a geometrical wear analysis of the inserts based on 3D scanning. Figure 5 shows the results of scanning the lower inserts made of both materials. In the analysis of the obtained results for the lower insert made of Hardox 600 steel (Figure 5a), we can state that, in the case of the lower part of the insert, the biggest loss of material, where the most intensive wear occurs, is present in the area responsible for the formation of the so-called wave. This can also be noticed in the case of the insert made of NC11LV steel (Figure 5b). In this area, for the insert made of Hardox, the wear reaches 2.31 mm. The biggest loss of material occurs along the whole edge on the side of the pug mill. In fact, on the whole length of this edge, the wear exceeds 2 mm, reaching the maximum of 2.49 mm. For the insert made of NC11LV steel, the wear mechanism is similar. In the remaining part of the insert, the highest wear is still visible on the edge, but it oscillates in the scope of 1.45 and 1.92 mm. The biggest loss of material for this tool reaches 2.26 mm.

**Figure 6:**
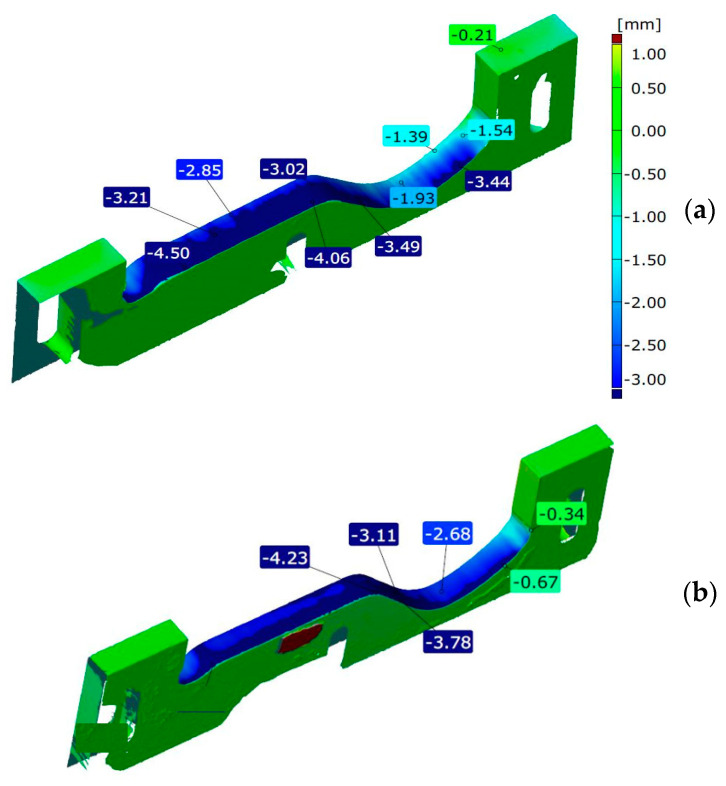
Results of scanning the upper tools made of steel: (**a**) Hardox 600 and (**b**) NC11LV.

For the upper inserts (Figure 6), the wear reaches a much higher level than in the case of the lower ones. This is due to the fact that the upper inserts can be adjusted and lowered, if the appropriate, acceptable shape of the tool is maintained of course. The highest wear for the insert made of Hardox steel can be observed on the side of the die of the pug mill. The most intensively worn area is visible on the fragment where the shape of the insert transfers from the “wave” into a flat area. In this section, the wear throughout the whole thickness of the material exceeds 3 mm. The wear reaches its maximum towards the end of the plate fragment and equals about 4.5 mm.

The wear of the upper insert made of NC11LV steel is also formed similarly to the insert analyzed before. Again, the highest loss of material is observed in the fragment shaping the flat part of the tile on the side of the pug mill. On this fragment, the wear varies between 2.7 and 4.3 mm.

### 3.4. Microstructural Tests

The chemical composition of the examined steels determined by the spectral method is presented in Table 1. The chemical composition of Hardox steel is in accordance with the composition declared by the steel’s manufacturer. According to the European standards, in respect to the chemical composition, the second material is in agreement with the grade X153CrMoV12 (1.2379 Steel). This steel is also known as NC11LV. The equivalent of this steel in the ASTM AISI standards [36] is grade D2. The main criterion classifying Hardox steels is their mean hardness in the as-delivered state, measured on the Brinell scale. Their chemical composition is selected in a way which enables control of the grade’s hardness, realized mainly through alloy element micro-additions as well as minimization of unfavorable additives (Table 1).

**Figure 7:**
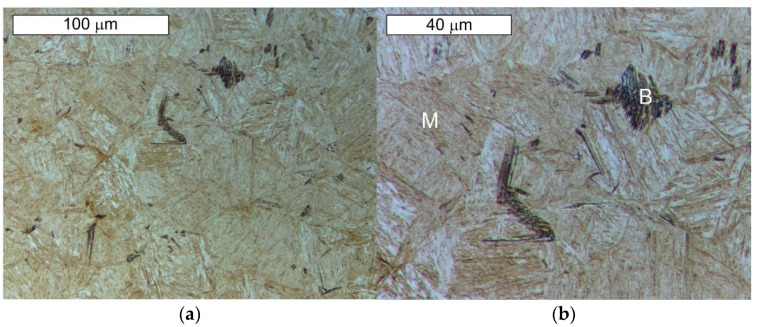
Microstructure of the examined Hardox steel obtained from sample 1: (**a**) visible tempered martensite (M) with locally occurring bainite (B); (**b**) magnified fragment of the area from Figure 7a. Light microscopy, etched state.

**Figure 8:**
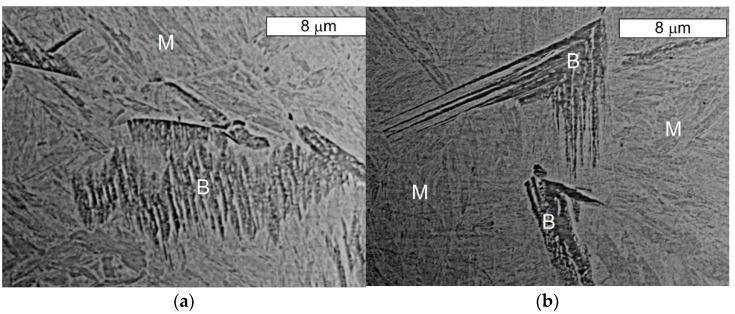
Microstructure of the Hardox steel obtained from sample 1: (**a**) visible bainite (B) with features typical for lower bainite on the tempered martensite matrix (M); (**b**) visible bainite (B) with features typical for upper bainite on the tempered martensite matrix (M). SEM, etched state.

**Figure 9:**
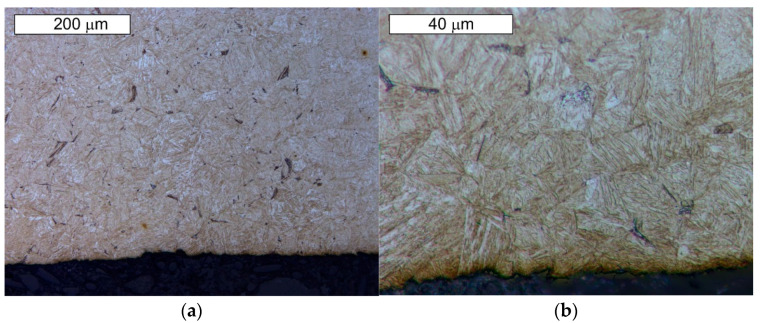
Material microstructure in the wear area on the cross-section of sample 1: (**a**) visible basically uniform surface fatigue; (**b**) magnified fragment of the area from Figure 9a. Light microscopy, etched state.

**Figure 10:**
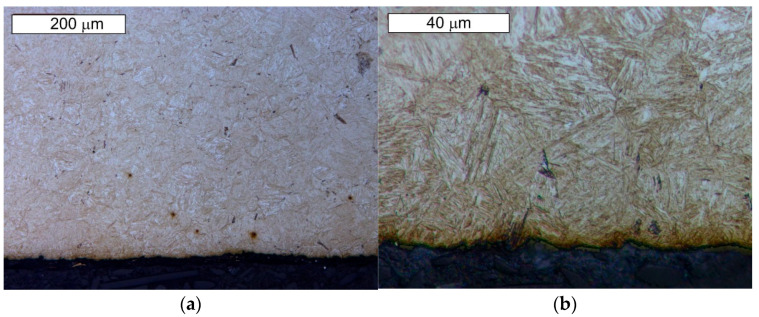
Material microstructure in the wear area on the cross-section of sample 2: (**a**) visible basically uniform surface fatigue; (**b**) magnified fragment of the area from Figure 10a. Light microscopy, etched state.

From the point of view of tool durability, its microstructure in the operation area of the surface layer is important. In the tests performed on the surface areas, the microstructure was identical for both examined samples. For that reason, the microstructural images are presented on the example of sample 1 taken from the location shown in Figure 1. The microstructure consisted of a tempered martensite matrix with upper and lower bainite (Figure 7 and Figure 8). The presence of locally occurring bainite in the martensite matrix indicates that the cooling is carried out in the range close to the critical quenching rate required to obtain a fully martensitic microstructure. It may also be related to the local heterogeneity of the chemical composition, which leads to a local reduction in the steel hardenability. It may also be indicated by a fairly significant amount of retained austenite, also occurring locally. A substantially uniform material wear was observed on the working surfaces. It is conditioned by the dominant volume fraction of martensite and a similar hardness of bainite and tempered martensite (Figure 9 and Figure 10). The results are presented for both samples taken from the locations shown in Figure 1.

**Figure 11:**
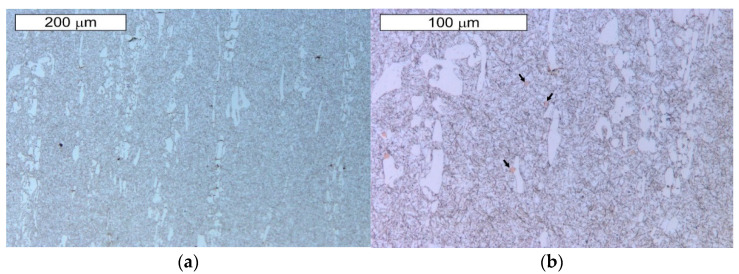
Microstructure of the examined steel X153CrMoV12 obtained from sample 1: (**a**) visible primary carbide precipitations arranged according to the plastic working direction in the martensitic matrix; (**b**) magnified fragment of the area from Figure 11a. Visible single nitride precipitations in the microstructure of the material (marked with an arrow). Light microscopy, etched state.

**Figure 12:**
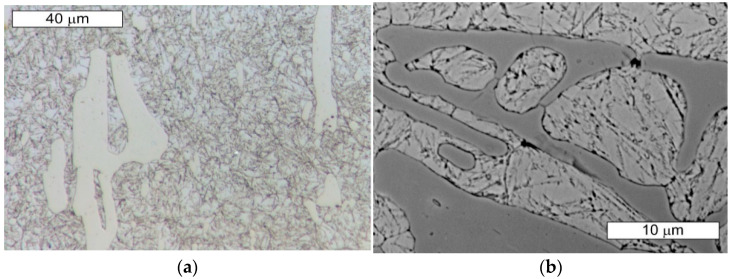
Magnified fragment of the area from Figure 11. Visible large primary carbide precipitations in the tempered martensite matrix; etched state: (**a**) light microscopy; (**b**) SEM.

**Figure 13:**
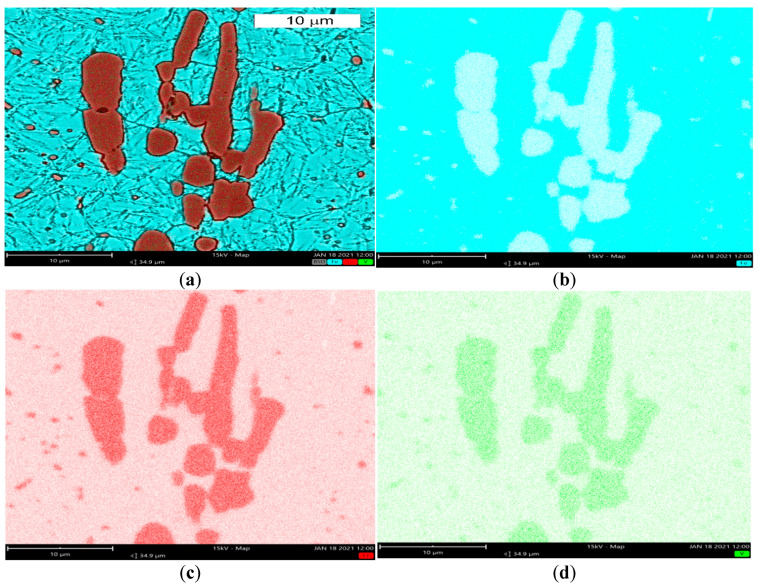
Element distribution in the microstructure area: (**a**) microscopic image with Fe, Cr and V distribution, (**b**) iron distribution, (**c**) chromium distribution, (**d**) vanadium distribution. SEM/EDX.

The microstructure of the tool steel in both examined areas of the element, 1 and 2, shown in Figure 1, was also identical (Figure 11 and Figure 12). Analogically to Hardox steel, the results of microscopic observations are presented on the example of sample 1 taken from the place shown in Figure 1. Steel belongs to the group of high-carbon and high-chromium materials. The high content of both of these carbide-forming elements leads to the formation of a large volume fraction of coarse primary carbides arranged according to the direction of the metal forming (Figure 11). Primary carbides are formed during the alloy solidification and they co-exist with austenite during plastic working. They play an important role in maintaining the high abrasion wear resistance of these materials. The presence of primary carbides in the microstructure of steel favors its higher hardness compared to Hardox steel. A high chromium content in these steels favors the precipitation of chromium carbides, which mainly form M_23_C_6_- and M_7_C_3_-type precipitations. The presence of vanadium in the chemical composition contributes to the possibility of MC-type carbide formation. Mohammed et al. [36] pointed to the fact that, at the austenitization temperature, only M_7_C_3_ carbides maintain their durability. The element distribution in the microstructure area is shown in Figure 13.

Chromium and vanadium were identified in the chemical composition of primary carbides. Another factor which ensures high hardness and abrasive wear resistance for these materials is their matrix.

**Figure 14:**
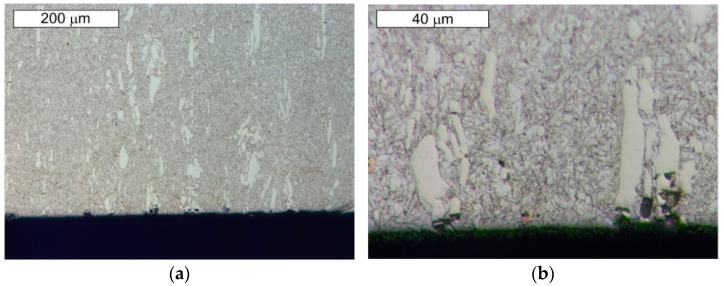
Material microstructure in the wear area of sample 1: (**a**) visible small spallings in the primary carbide area; (**b**) magnified fragment of the area from Figure 14a. Cross-section. Light microscopy, etched state.

**Figure 15:**
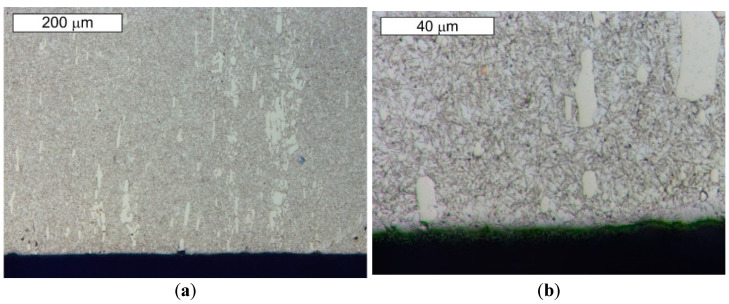
Material microstructure in the wear area of sample 2: (**a**) visible small spallings in the primary carbide area; (**b**) magnified fragment of the area from Figure 15a. Cross-section. Light microscopy, etched state.

These steels, after quenching, undergo tempering at 180 °C, with the aim to obtain tempered martensite. During the tempering process, the needle-shape character of martensite becomes less profound, and, as a result of redistribution of the carbon atoms, fine, uniformly distributed secondary carbides precipitate, which, at this tempering stage, are coherent with the matrix. Secondary precipitation mainly occurs in the case of those carbides which dissolved during the austenitization. In the material microstructure, single orange nitride precipitations with irregular shapes were also observed (Figure 11b). Microscopic observations on the cross-sections were also performed on the edges subject to wear of both examined samples. No differences in the wear character of the edges of the samples collected from areas 1 and 2 were observed. In the microscopic area, the formation of small spallings in the primary carbide precipitation areas was recorded, yet they did not propagate into deeper material areas (Figure 14 and Figure 15).

The large number of hard carbides (mainly Cr) observed in the microstructure of this steel may result in increased hardness in relation to Hardox steel.

### 3.5. Analysis of Materials’ Topography

**Figure 16:**
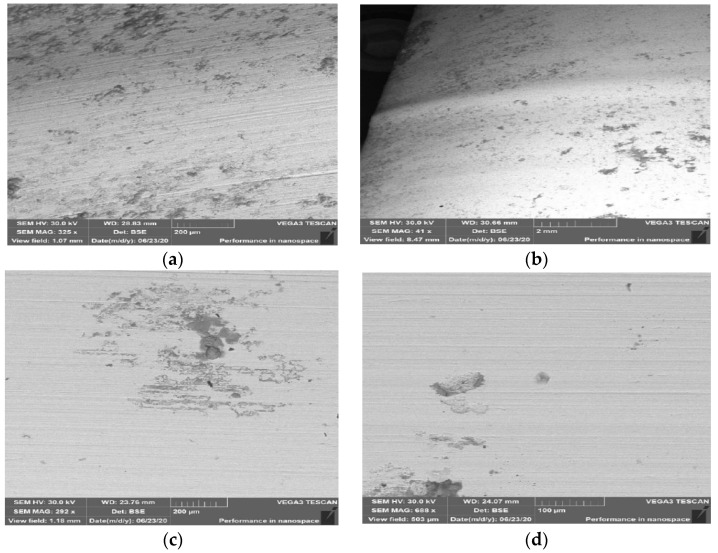
Surface topography of samples made of Hardox 600 steel: (**a**) external surface for sample from area 1; (**b**) core for sample from area 1, for lower tool; (**c**) external surface for sample from area 2; (**d**) core for sample from area 2, for lower tool. SEM.

The examined samples underwent an analysis of the material’s topography for both types of steel in order to reveal defects formed in the areas of contact between the tool and the band during its operation. Figure 16 shows the results of the surface topography tests for samples made of Hardox 600 steel. In the analysis of the topography images (Figure 16a,b) of the surface of the lower half of the insert made of Hardox 600 steel, we can observe numerous scratches. The scratches formed on the surface are the effect of abrasion wear. The direction of the scratches on the external surface of the insert is in accordance with the movement of the extruded band of the material. In turn, on the upper tool, we can see numerous linear surface scratches. Further, losses of material are visible. The presence of this defect can be caused by the pulling out of the material’s surface layer as a result of structure disarrangement by the hard fractions of the processed mass. Of course, leaving such defects on the working surface of the insert can result in the presence of defects on the surface of the manufactured band, which can then become critical for the ready product.

**Figure 17:**
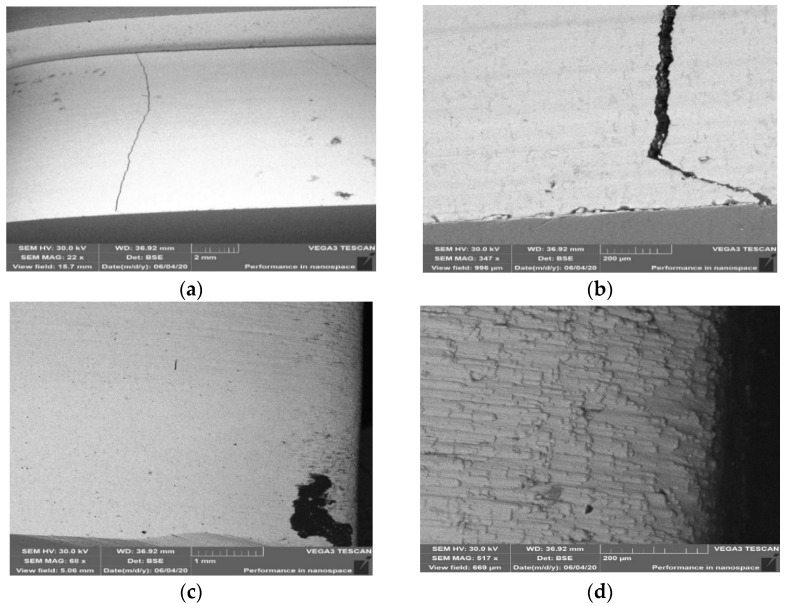
Topography of the surface for samples made of steel NC11LV: (**a**) external surface for sample from area 1; (**b**) core for sample from area 1, for lower tool; (**c**) external surface for sample from area 2; (**d**) core for sample from area 2, for lower tool. SEM.

For samples made of NC11LV taken from area 1 shown in Figure 1, in the lower half of the insert, we can observe cracking of the material (Figure 17a,b), as well as point traces of indentations and growths on the surface (Figure 17c). The cracking occurred parallel to the edge shaping the clay band in the surface layer.

The formed cracks on the surface of this material were probably caused by the effect of hardening, which resulted in increased hardness as well as brittleness of the surface layer. Further operation of the insert with such a defect can lead to the propagation of cracks or spalling of the material as a result of contact with the harder fractions present in the treated mass and a rapid acceleration of wear. On the edge onto which the treated material presses during the machine’s operation, we can observe plastic deformation (Figure 17c). The phenomenon of plastic deformation of the abraded material did not occur on the lower half of the insert, where the material’s abrasion mechanism is mainly based on ridging and micromachining. On a sample collected from area 2 from the upper half of the insert (Figure 4d), we can notice the material’s wear as a result of the operation of an abradant in the form of mass used to manufacture the ceramic tiles. Such defects revealed at too high a degree are connected with the necessity to regenerate such a tool in order to enable its further operation.

### 3.6. Hardness Measurements

**Figure 18:**
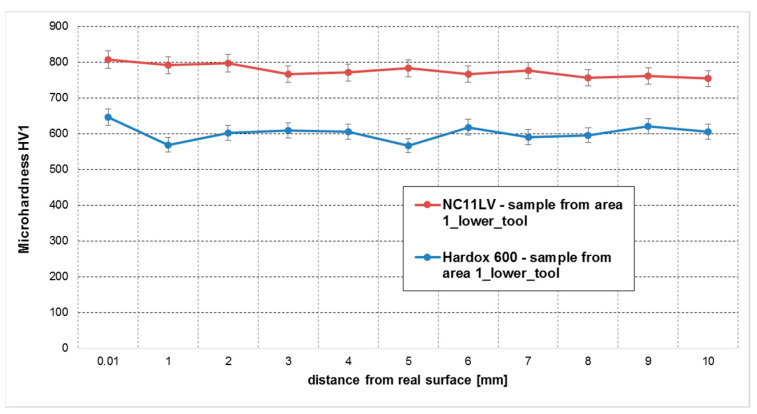
Hardness measurements at a distance from the working surface for area 1 (steels NC11LV and Hardox).

**Figure 19:**
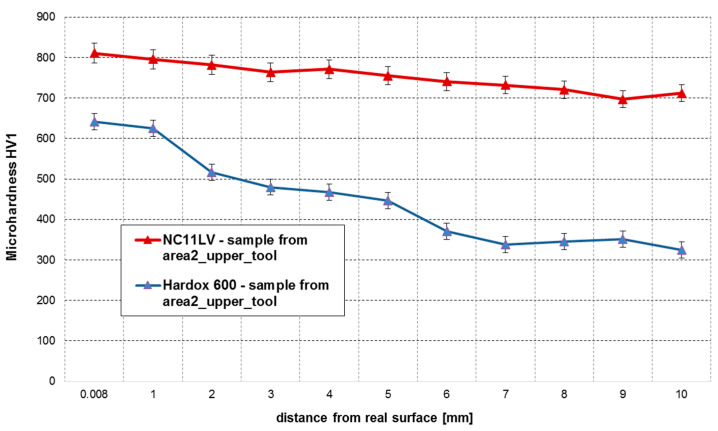
Hardness measurements at a distance from the working surface for area 2 (NC11LV and Hardox 600 steels).

In order to verify the properties of the examined materials, a hardness measurement was carried out. The figures below compile in diagrams the hardness measurement results by comparing the hardness for both materials on the lower insert (Figure 18) and the upper insert (Figure 19).

In the case of hardened NC11LV steel, we can observe that the material’s hardness drops slightly with the increasing distance of the measurement points from the edges of the insert. The hardness in the surface layer of the inserts reaches the level of 807 HV, which can point to reinforcement of this steel as a result of high pressures coming from abrasion. We also have to consider the possibility that a slightly higher hardness is connected with a higher value of internal stresses occurring after hardening, which result from a more rapid quenching taking place in the surface areas. Further into the material, the hardness stabilizes at the level of about 770 HV. A sample collected from the lower part of the insert made of Hardox 600 steel is also characterized by a stabilized hardness, regardless of the distance from the edge shaping the band. In the examined sample, the hardness oscillates in the range from 566 to 646 HV1. In the case of the insert from this material (Hardox), the obtained results are in accordance with the data provided by the manufacturer.

In the analysis of the results obtained for the upper tools (Figure 10), we can notice that, in the case of the insert made of NC11LV steel, again, the maximal hardness reaches the level of over 800 HV1. The hardness of this insert, starting from the working surface, begins to drop to 700 HV1 towards its inside. In the case of Hardox 600, very big discrepancies between the particular hardness measurements were observed in a sample collected from the upper half of the insert. In the layer by the shaping area, the recorded hardness values were at the level of 641 HV1, and they gradually dropped with the increasing depth of the material to the level of 325 HV1.

The cause of the occurrence of such a drop could have been the fact that the upper tools made of both materials are shifted during the process, so that, through their regulation (lowering), a specific thickness of the band can be maintained. The use of an angle grinder to cut the samples can have a negative effect on the material and lower its hardness. A more intense decrease in hardness in the case of Hardox compared to NC11LV may be due to the fact that the tool steel (NC11LV) partially strengthens during cold work, due to the deformation of the clay strand from the pressure.

### 3.7. The Impact Toughness

The impact strength tests were performed on samples with a V-notch made of Hardox 600 and NC11LV steels, according to EN 10045-1. Two tests were performed for each tool for the upper and lower inserts for both materials at a temperature of 20 and 40 °C. The temperatures were selected to relate the obtained results to the literature data (20 °C) and to determine the effect of the elevated temperature (40 °C), which was observed in the process of extruding the strand at the edges of the tools. The obtained results are summarized in Table 2.

Hard carbides and the carbide/matrix interface exhibit a lower fracture propagation resistance than the martensitic matrix, which dissipates some of the energy released during the fracture due to plastic deformation. Large carbide particles have a strengthening effect as they limit the plastic deformation of the matrix. Consequently, the presence of hard and non-deformable carbides increases the hardness of the tool steel but reduces its impact toughness.

The obtained results show that the determined values of impact toughness at the temperature of 20 °C for both materials are in line with the literature data and those provided by the manufacturers. There were also no significant differences in the impact toughness values for the upper and lower tools. However, the impact toughness results obtained at higher temperatures (40 °C) are slightly higher, and the increased operating temperature of the inserts has a positive effect on their durability. Therefore, it seems that a slight increase in the extrusion speed, acceptable in terms of the technology and technical parameters of machines, should positively affect the extension of their operation time.

## 4. Summary and Discussion

The study performed a comparative analysis of the wear for selected tools used to form clay bands in the production of ceramic roofing tiles. The conducted tests and measurements in the aspect of abrasion resistance made it possible to point to a material more suitable during the shaping of a clay band. On this basis, the following conclusions were drawn:

1. A longer durability was exhibited by the inserts made of NC11LV steel compared to the inserts made of Hardox 600 steel. In the performed analysis, both types of inserts demonstrated a similar level of wear, which, in the most critical points, reached about 4.5 mm. However, the insert made of NC11LV steel worked over a much longer period without regeneration, equaling 912 h, whereas the insert made of Hardox 600 steel operated for the time of 384 h. This means that the inserts made of NC11LV steel worked over a period 58% longer than the inserts made of Hardox 600 steel. This can be related to the microstructure of the material. The carbide precipitations delay the propagation of the initiated crack by deflecting or branching the crack path, which can extend the tool service life.
2. On the performed 3D scans of the worn inserts, it is easy to observe that the inserts wear off non-uniformly. Regardless of the material, the upper part of the insert is characterized by a much higher degree of wear than the lower part. The cause of this non-uniform wear of the inserts is their construction as well as the system of mounting the inserts to the head of the pug mill. The vertical system in which the inserts are mounted on the press determines the regulation only with the upper insert. Therefore, the system should be modernized in such a way that it is possible to uniformly regulate both the upper and lower inserts.
3. The maximal hardness of NC11LV steel reached about 800 HV1, whereas the hardness of Hardox 600 steel reached its maximum at about 650 HV1. Such a hardness level for tools made of NC11LV was obtained owing to the applied heat treatment and the presence of hard precipitates of primary carbides.
4. In the case of impact toughness, much higher KV values were obtained for Hardox 600 steel-above 70 J at a temperature of 20 °C and about 80 J at a temperature of 40 °C, which is also the working temperature of the tools. For NC11LV steel, the obtained KV values are over 4 times lower, which may have an adverse effect in the case of significant dynamic forces. The lower value of impact toughness is associated with the presence of carbides in the microstructure, which have a strengthening effect by reducing the plastic deformation of the matrix.
5. Comparing the time worked over by both sets of inserts and the costs related to their manufacture, we can see that the inserts made of NC11LV steel are also more advantageous, which, despite a 42% higher price, can operate throughout a much longer period of time. Owing to this, the necessity of replacing these elements becomes limited and thus the number of standstills of the production line becomes reduced. The inserts made of NC11LV steel were also characterized by a much higher hardness in the surface layer than the inserts made of Hardox 600 steel.

## 5. Conclusions

Based on the obtained results, it seems that, in the case of the described industrial application, the process of extruding a strand of clay onto ceramic tiles is a stumbling block, and so the use of a much harder, yet less impact-resistant and cheaper NC11LV steel for tools is justified. A comprehensive comparative analysis for tools made of both steels shows that, apart from better impact parameters, tools made of NC11LV steel withstood less than a 3 times longer service life, while maintaining the required geometric conditions and surface quality. One should also take into account the aspect of machine downtime and the need for more frequent tool changes, and thus a lower productivity and higher unit production costs. 

The directions of further research will be a further optimization of inserts made of NC11LV steel in the scope of thermo-chemical surface hardening treatment as well as other aspects, and in the case of inserts made of Hardox steel, protective coatings or other heat treatment variants could be applied, so that the selected parameters can provide the possibility to reach the highest possible abrasion resistance. 

## Figures and Tables

**Table 1:** Chemical composition of NC11LV and Hardox 600 steels (%).

Chemical Elements/Materials	C	Si	Mn	P	S	Ni	Mo	B	Cr	Cu	V	W
Hardox600	0.41	0.51	1.28	0.01	0.01	0.21	0.66	0.003	1.11	-	-	0.18
NC11LV	1.64	0.21	0.37	0.03	0.02	0.22	0.87	-	11.06	0.31	0.72	-

**Table 2:** The impact strength results for Hardox 600 and NC11LV steels (KV in J).

Tools/Materials	Upper Insert 20 °C	Lower Insert 20 °C	Upper Insert 40 °C	Lower Insert 40 °C
Hardox 600	77/79	74/77	81/82	78/80
NC11LV	15/17	14/15	21/22	19/21

## Data Availability

Data sharing not applicable.
